# Role of the Basolateral Na^+^/H^+^ Exchanger-2 (NHE2) in Ionocytes of Seawater- Acclimated Medaka (*Oryzias latipes*)

**DOI:** 10.3389/fphys.2022.870967

**Published:** 2022-03-24

**Authors:** Sian-Tai Liu, Jiun-Lin Horng, Li-Yih Lin

**Affiliations:** ^1^Department of Life Science, School of Life Sciences, National Taiwan Normal University, Taipei City, Taiwan; ^2^Department of Anatomy and Cell Biology, School of Medicine, College of Medicine, Taipei Medical University, Taipei City, Taiwan

**Keywords:** salt secretion, acid-base balance, medaka, ionocyte, Na^+^/H^+^ exchange

## Abstract

Ionocytes in the skin and gills of seawater (SW) fishes are responsible for acid-base regulation and salt secretion. Na^+^/H^+^ exchangers (NHEs) are considered the major acid (H^+^)-secreting transporters in ionocytes of SW fishes. However, the subcellular localization and function of a specific NHE isoform (NHE2) have never clearly been revealed. In this study, we cloned and sequenced NHE2 from an SW-acclimated medaka (*Oryzias latipes*) and examined its functions in medaka embryos. A phylogenetic analysis showed that the evolutionary relationships of mammalian NHE2 and NHE4 are close to those of fish NHE2. A gene structure analysis showed that tetrapod NHE4 might be a tandem duplication of fish NHE2. Immunohistochemistry with a medaka-specific antibody localized NHE2 to the basolateral membrane of ionocytes. Lost-of-function experiments with photo-activated morpholino oligonucleotides showed that both H^+^ and Cl^–^ secretion by ionocytes were suppressed in NHE2-knockdown embryos, suggesting that the basolateral NHE2 facilitates acid and salt secretion by ionocytes of medaka in seawater.

## Introduction

Acidification of both marine and fresh water is a major environmental problem, and it poses a global threat to aquatic animals including fishes. Understanding the molecular and cellular mechanisms for acid-base regulation in fishes can establish the fundamental knowledge to evaluate the impacts of environmental acidification on fishes. Acid-base regulation in fishes is mainly achieved by gills and skin (Evans et al., 2005; Hwang et al., 2011). In gill and skin epithelium, ionocytes (also called mitochondria-rich cells or chloride cells) play major roles in ion and acid-base regulation (Yan and Hwang, 2019). It is generally accepted that sodium (Na^+^) and hydrogen (H^+^) exchangers (NHEs) are the major transporters of acid (H^+^) secretion by ionocytes (Hwang and Chou, 2013; Liu et al., 2013). Several NHE isoforms (NHE1, NHE2, NHE3, NHE5, NHE6, NHE7, and NHE8) were found in fishes, (Hu et al., 2013; Yan and Hwang, 2019); and NHE1, NHE2, and NHE3 were found in the gills of fishes (Claiborne et al., 1999, 2002; Edwards et al., 2005). In freshwater (FW) fishes, NHE3 at the apical membrane was demonstrated to be the major isoform for acid secretion by ionocytes (Yan et al., 2007; Inokuchi et al., 2008; Wu et al., 2010).

In seawater (SW) fishes, two NHE isoforms, NHE2 and NHE3, were identified in ionocytes of gills. In Japanese eel (*Anguilla japonica*) and mangrove rivulus (*Kryptolebias marmoratus*), NHE3 was localized to the apical membrane of ionocytes (Cooper et al., 2013; Seo et al., 2013). In medaka (*Oryzias latipes*) acclimated to SW, NHE3 was localized to apical membranes of ionocytes and demonstrated to be involved in acid secretion (Liu et al., 2013, 2016). Compared to NHE3, however, the localization and function of NHE2 in ionocytes of SW fish are still uncertain. Several studies showed that messenger (m)RNA and protein expressions of NHE2 were stimulated by acidic water or infusion of HCl, suggesting that NHE2 is involved in acid secretion by gills (Claiborne et al., 1999; Tresguerres et al., 2005; Catches et al., 2006).

Medaka is highly adaptable to different salinities, is amenable to genetic manipulation, and is suitable for studies of organogenesis (Wittbrodt et al., 2002; Takeda and Shimada, 2010). Our previous studies demonstrated that medaka embryos are an ideal model for the functional study of ion transport and acid-base regulation by skin ionocytes (Wu et al., 2010; Shen et al., 2011; Liu et al., 2013, 2016). Previously, we found that both NHE2 transcripts and NHE3 proteins were localized at the same ionocytes in SW-acclimated medaka embryos (Liu et al., 2013), and demonstrated that H^+^ secretion by ionocytes was linked to Cl^–^ secretion (Liu et al., 2016). However, the protein localization and function of NHE2 in SW-type ionocytes are still unclear. Is NHE2 also located on the apical membrane of ionocytes? If so, what is the functional difference between NHE2 and NHE3 in acid secretion? We attempted to answer these questions in this study. First, we cloned and sequenced NHE2 (*slc9a2*) from gills of medaka, and analyzed the evolutionary relationships among fish and mammalian NHE paralogs (NHE1∼9) with a phylogenic tree analysis. We analyzed the gene structures of NHE2 and NHE4 orthologs to clarify evolutionary relations between NHE2 and NHE4. In addition, we also collected the deduced amino acid sequences of NHE2 and compared differences between fishes and mammals. Second, a specific antibody was generated to localize NHE2 in ionocytes. Finally, we knock down the gene expression of NHE2 in medaka embryos by using photo-activated morpholino oligonucleotides (photo-MOs), which can suppress the gene expression at a specific stage of embryos (Tallafuss et al., 2012). Functional changes (H^+^ and Cl^–^ secretion) were analyzed *in vivo* with an electrophysiological technique following our previous studies (Wu et al., 2010; Shen et al., 2011; Liu et al., 2016).

## Materials and Methods

### Medaka Husbandry and Seawater Acclimation

The animal husbandry and experimental protocols of the present study were reviewed and approved by the Animal Care and Utilization Committee of National Taiwan Normal University. Adult medaka (*Oryzias latipes*) were reared in 1200 (length) × 450 (width) × 600 (height)-mm FW tanks equipped with water filters. We refreshed a quarter of the tank water every week. The water temperature of the tanks was controlled to 28°C by a thermal controller and the photoperiod of the animal room was controlled 14 h of light and 10 h of the dark. Fish were fed at least twice a day. In the morning of ten to ten-thirty, the fertilized eggs were collected from the belly of the female medaka. For SW acclimation, the collected eggs were directly transferred to Petri dishes containing 30 ppt SW and incubated at 28°C. We added the appropriate amounts of sea salt (Instant Ocean, Aquarium System, Mentor, OH, United States) to FW to prepare SW. SW-acclimated adult medaka were obtained by transferring adult fish to 30 ppt SW tanks for 2 weeks.

### Phylogenetic Tree and Genomic Sequences Analysis

Coding sequences of known and predicted medaka NHE homologs were obtained from GenBank and Ensembl databases. For the phylogenetic analysis of NHEs candidates, the multiple sequence alignment program “ClustalW2” (European Molecular Biology Laboratory, Heidelberg, Germany) was used to compare the deduced amino acid sequence of medaka NHE homologs with all known data available in the European Institute of Bioinformatics (EBI) database and were subjected to phylogenetic inferences using the Neighbor-joining (NJ) method. The MEGA 6.0 (Molecular Evolutionary Genetics Analysis Version 6.0) software was used to perform 1,000 bootstrap replicate analyses. Based on the assemblies of the Ensembl Genome Browser, the physical gene map of the verified *slc9a2* (NHE2) and *slc9a4* (NHE4) loci was scaled. Genes located up-and downstream of *slc9a2* (NHE2) and *slc9a4* (NHE4) genes in these loci were compared to genomes of fish, amphibians, reptilian and mammalian to determine the highest score.

### Preparation of RNA and Reverse-Transcription Polymerase Chain Reaction

mRNA levels of NHE2 in different tissues (brain, gills, eyes, heart, liver, intestines, spleen, kidneys, muscles, and fins) of SW-acclimated adult medaka were determined using an RT-PCR. Tissues isolated from three to seven individuals were pooled as one sample. We used Trizol reagent (Invitrogen, Carlsbad, CA, United States) to homogenize all samples according to a volume ratio of 1:10 (sample: Trizol). After homogenizing, 0.1 ml of 1-bromo-3-chloropropane (BCP) solution (Sigma-Aldrich, St. Louis, MO, United States) was added to each sample. After centrifugation at 4°C and 14,000 rpm for 30 min, the supernatant was extracted and taken into the same volume of 100% isopropanol for RNA precipitation. Subsequently, total RNA was purified and DNA contamination was removed by a MasterPure™ RNA Purification Kit (Epicentre Biotechnologies, Madison, WI, United States). After purifying total RNA, the amount and the quality of total RNA were determined by a NanoDrop 1000 spectrophotometer (Thermo Scientific, Wilmington, DE, United States). The integrity of total RNA was checked by electrophoresis through an RNA-denaturing gel.

For the reverse-transcription reaction, 3 μg of total RNA was added into a final volume of 13 μl containing 1 μl of 10 mM dNTPs, 1 μl of 50 mM oligo(dT)_20_ at 65°C for 5 min, and then the samples immediately moved onto the ice for at least 1 min. After that, each sample was supplemented to a final volume of 20 μl containing 5 mM dithiothreitol (DTT), 40 units of an RNase inhibitor (RNAaseOUT) and 200 units of SuperScript IV (SSIV) (Invitrogen, Carlsbad, CA, United States), and then incubated at 55°C for 15 min. For PCR amplification, 0.5 μl of complementary (c)DNA (dilute 1:200 by diethylpyrocarbonate water) from various tissues, including the brain, gills, eyes, heart, liver, intestines, spleen, kidneys, muscles and fins, were used as a template in a 25-μl final reaction volume containing 1 μl of 10 mM dNTP, 1 μl of 40 units of Gen-Taq polymerase (Genemark, Taipei, Taiwan), and 1 μl of 10 mM of NHE2 primers (forward: 5′-ATCGTCTGTTGTGCCCTC-3′; reverse: 5′-CAGTTCCACTCGTGCTCT-3′).

### Morpholino Oligonucleotides Design and Microinjection

An antisense morpholino-modified oligonucleotides (MOs; Open Biosystems, Huntsville, AL) with the sequence 5′-TTCTCGTGGACCGAGTACATGATGC-3′ was used to target −6 to + 19 of the coding region of medaka NHE2; a sense photo-activated (photo)-MOs (Open Biosystems, Huntsville, AL), 5′-GCATCATGTACT-photo-GGTCCAC-3′, was used to target normal MOs with the middle of the photo-sensitive subunit. Standard control oligonucleotides (5′-ATCCATCTTGTGTGTTAGAAAACTG-3′; Gene Tools, Philomath, OR, United States) were used as the control MO, based on its no target and no significant biological activity.

The MOs were resuspended in 1X Danieau’s solution, stored at −20°C as a stock solution. Before use, we diluted the stock to desired concentrations (0.5, 1.0, 2.0, or 4.0 ng/embryo). In the present study, both MOs (0.5 ng/embryo) and photo-MOs (0.6 ng/embryo) were incubated at 50°C for 10 mins. Subsequently, both MOs and photo-MOs were co-injected into embryos at one to the two-cell stage by a gas-driven microinjection apparatus (ASI, Eugene, OR). After injection with both NHE2 MOs and photo-MOs, 5-day post-fertilization (dpf) embryos were stimulated by UV light (365 nm) for 1 min from UV Lightbox (Gene Tools, Philomath, OR, United States), and then 8-dpf embryos were sampled to measure proteins expression. The 5-dpf embryos not stimulated with UV light (365 nm) served as the control photo-MOs.

### Immunohistochemistry

We collected 8-dpf embryos and fixed them in 4% paraformaldehyde (PFA) at 4°C overnight. The protocol for NHE2 IHC followed by that of a previous report (Inoue and Wittbrodt, 2011). For the immunoreaction, a polyclonal antibody against the domain (VAYPGRRSRFGNRSS; Genomics, Taipei, Taiwan) of medaka NHE2 (diluted 1: 300) and goat anti-rabbit immunoglobulin G (IgG) conjugated with Alexa Fluor 488 (Molecular Probes, Carlsbad, CA) (diluted 1: 500) were, respectively, used as the primary and secondary antibody, respectively. For Na^+^/K^+^-ATPase (NKA) staining, an α5-monoclonal antibody against the α-subunit of the avian NKA (Developmental Studies Hybridoma Bank, University of Iowa, Ames, IA) was used to detect NKA. The protocol was conducted as described in previous reports (Wu et al., 2010; Liu et al., 2013). Finally, images were captured by using a fluorescence microscope (Axioplan 2 Imaging, Carl Zeiss Oberkochen, Germany).

### Western Blotting

Ten 8-dpf embryos were collected as one sample and washed twice with phosphate-buffered saline (PBS). Afterward, homogenization buffer (100 mM imidazole, 5 mM EDTA, 200 mM sucrose, and 0.1% sodium deoxycholate) (with the pH adjusted to 7.6) with 10 units of protease inhibitors (Roche, Indianapolis, IN, United States) was used to homogenize all samples. After centrifuging at 4°C and 10,000 rpm for 10 min, the supernatant was extracted for analysis by using BCA protein assay reagents (Pierce Chemical Company, Rockford, IL, United States). Each sample (equivalent to a volume of 20 μg protein) was incubated at 95°C for 10 min for denaturing and then subjected to 10% sodium dodecyl sulfate (SDS)-polyacrylamide gel electrophoresis (PAGE). After electrophoresis, the gel was transferred to polyvinylidene difluoride membranes (Millipore, Billerica, CA, United States). For the blocking reaction, the membranes were transferred to 5% non-fat milk at room temperature for 2 h. Afterward, blots were incubated with a rabbit anti-medaka NHE2 polyclonal antibody (diluted 1:1000) and a rabbit anti-human β-actin (Abcam, Tokyo, Japan, diluted 1:1000) at 4°C overnight. Signals of blots were enhanced with a chemiluminescence system (Millipore, Billerica, CA, United States). The membranes were incubated with goat anti-rabbit IgG (H + L) HRP (dilute 1:10000; Invitrogen, Carlsbad, CA, United States). The ImageQuant 4000 system (GE Healthcare, Buckinghamshire, United Kingdom) was used to capture the clear images for analysis. Finally, we quantitated the intensities of immunoreactive bands using ImageJ software^1^.

### Measurement of H^+^ Flux and Cl^–^ Flux at Ionocytes

The scanning ion-selective electrode technique (SIET; also called the non-invasive micro-test technique, NMT) was used to measure H^+^ and Cl^–^ secretion by skin ionocytes of medaka larvae. H^+^-selective and Cl^–^-selective microelectrodes were prepared as described in previous studies (Shen et al., 2011; Liu et al., 2016). The signal of the microelectrode was connected to the main amplifier through an Ag/AgCl wire electrode holder and preamplifier (Younger United States, Amherst, MA, United States). The movement and positioning of the microelectrodes were performed with a three-dimensional (3D) positioner (Younger United States) driven by a step motor. Imfluxes V2.0 software (youngerusa.com; xuyue.net) was used to control the system and calculate ion fluxes.

### Statistical Analysis

Data are presented as the mean ± standard error of the mean (SEM). Student’s unpaired *t*-test (two-tailed) was used for comparisons of the means of two groups. The significance level was set to *p* < 0.05.

## Results

### Phylogenetic Analysis, Sequence Identity, and Sequence Analysis of Medaka NHE2

**FIGURE 1:**
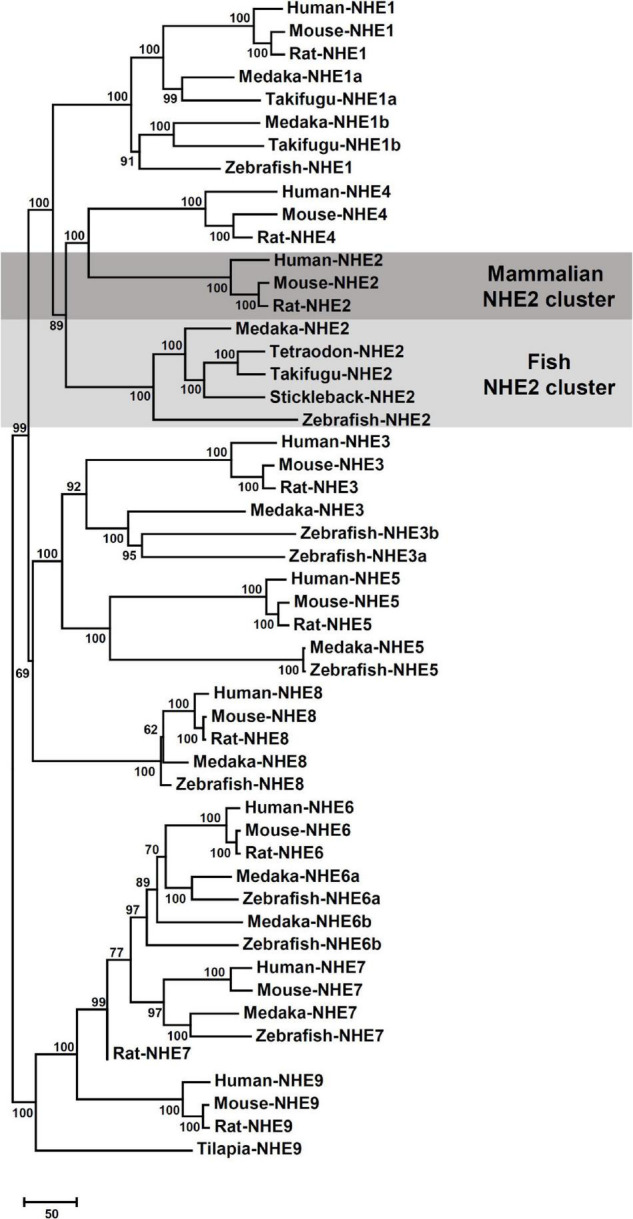
Phylogenetic tree based on comparisons of Na^+^/H^+^ exchanger (NHE) sequences. Putative sequences of amino acids in medaka (*Oryzias latipes*) and other species were collected from NCBI and Ensembl databases (listed in Supplementary Table 1). The Neighbor-joining method was used to build the phylogenetic trees of NHEs and confirmed by 1000 bootstraps. The unit of the scale bar represents the number of substitutions per site.

**FIGURE 2:**
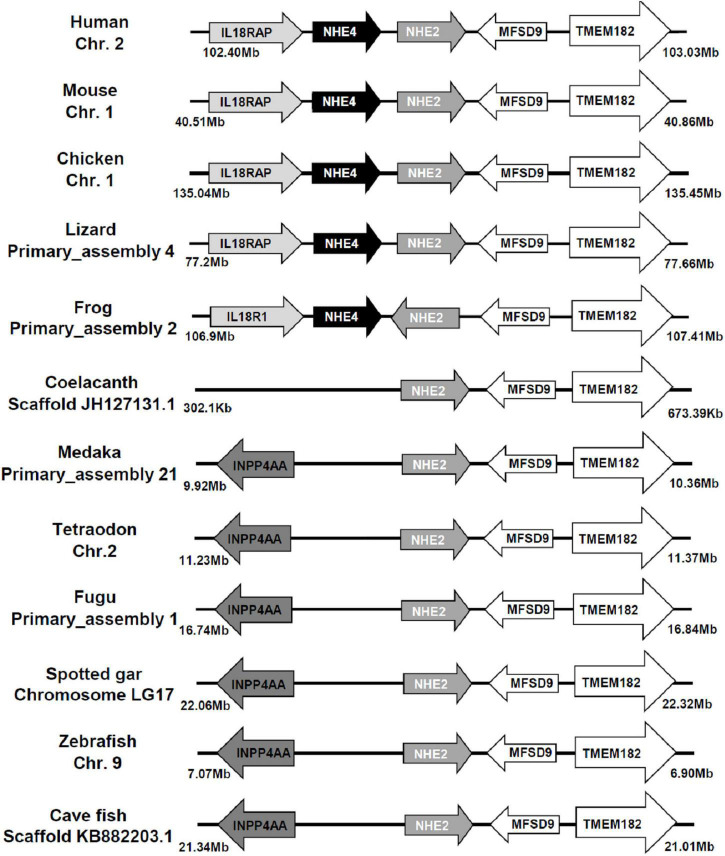
Gene structures of Na^+^/H^+^ exchanger 2 (NHE2) and NHE4 orthologs. The distance of the genomic region is presented on both sides. The arrow indicates the direction of the gene. Both NHE2 and NHE4 orthologs and their neighboring transcripts were obtained from NCBI and Ensembl databases (as described in Supplementary Table 1). Chr., chromosome; IL18RAP (IL18R1), interleukin-18 receptor accessory protein; INPP4AA, inositol polyphosphate-4-phosphatase type I; MFSD9, major facilitator superfamily domain containing 9; TMEM182, transmembrane protein 182.

The sequence of medaka NHE2 (ENSORLG00000012399) was predicted using the Ensembl database and was further cloned and sequenced. The NJ method was used to generate the phylogenetic tree of protein sequences of NHEs from human (*Homo sapiens*), mouse (*Mus musculus*), rat (*Rattus norvegicus*), medaka (*Oryzias. latipes*), zebrafish (*Danio rerio*), tilapia (*Oreochromis niloticus*), stickleback (*Gasterosteus aculeatus*), and pufferfish (*Takifugu rubripes* and *Tetraodon nigroviridis*). The phylogenetic analysis demonstrated that the eight members of medaka NHEs (NHE1, NHE2, NHE3, NHE5, NHE6, NHE7, NHE8, and NHE9) were classified into different groups and clustered with orthologues from mammals (Figure 1). According to the phylogenetic analysis, the fish NHE2 clade was most closely related to the mammalian NHE2 and NHE4 clades. Further, gene arrangements in the genomic regions encompassing NHE2 and NHE4 were compared. Results showed that NHE2, TMEM182, and MFSD9 were evolutionarily located on the same chromosome from fish to mammals except for INPP4AA (Figure 2). The *NHE4* gene occurred beside *NHE2* on the same chromosome of amphibians, reptiles, birds, and mammals but not in fishes, suggesting that *NHE2* duplication had occurred in the chromosome of amphibians and that had formed *NHE4*.

**FIGURE 3:**
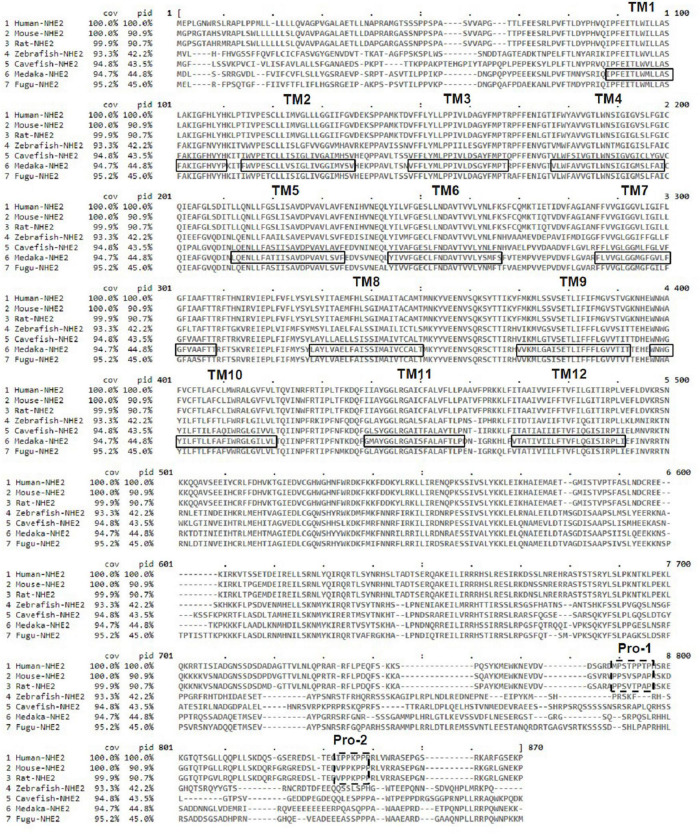
Alignment of the deduced amino acid sequences of Na^+^/H^+^ exchanger 2 (NHE2) of medaka and other species. The solid line and dotted lines, respectively, mark the postulated transmembrane (TM) domains of NHE2 and proline-rich sequences (Pro-1 and Pro-2).

The putative transmembrane (TM) protein prediction of medaka NHE2 was predicted using TMHMM (server v. 2.0)^2^ (Figure 3). Results showed that 12 TMs (black line) were found in medaka NHE2. In addition, compared to amino acid sequences of mammalian NHE2, we found that fish NHE2 lacked the two proline-rich sequences (Pro-1 and Pro-2) (dashed line) (Figure 3). It was demonstrated that Pro-1 and Pro-2 are necessary for appropriate subcellular targeting of rat NHE2 (Chow et al., 1999).

### NHE2 (*slc9a2*) mRNA Expression in Various Medaka Tissues

**FIGURE 4:**
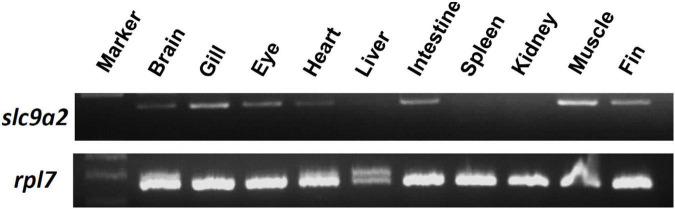
Distribution of *slc9a2* in various tissues of medaka. *slc9a2* transcripts were detected in adult medaka by an RT-PCR. *rpl7* was used as an internal control.

To examine the distribution of *slc9a2* in medaka tissues, mRNA expression of *slc9a2* in various tissues including the brain, gills, eyes, heart, liver, intestines, spleen, kidneys, muscles, and fins, were examined by an RT-PCR. The result showed that *slc9a2* was abundantly expressed in the gills, intestines, and muscles (Figure 4). In contrast, *slc9a2* expression was quite weak in the liver, spleen, and kidneys (Figure 4). In this study, *rpl7* mRNA was used as an internal control.

### Immunolocalization of NHE2 in Embryonic Ionocytes

**FIGURE 5:**
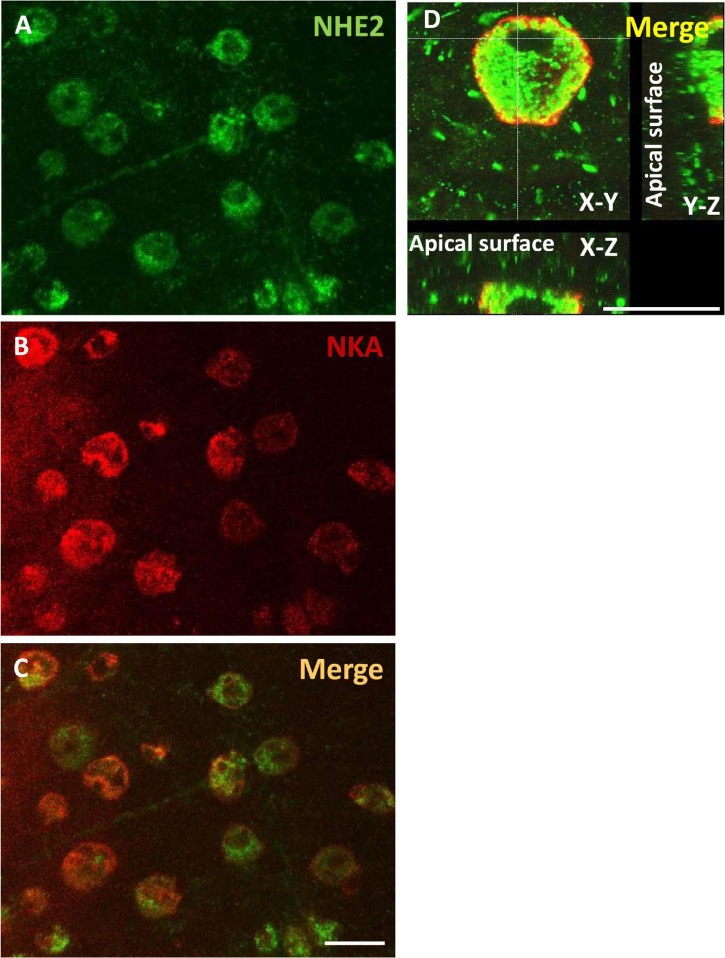
Localization of Na^+^/H^+^ exchanger 2 (NHE2) in the skin of seawater (SW)-acclimated medaka embryos. Immunocytochemistry of NHE2 **(A)** and Na^+^-K^+^-ATPase (NKA) **(B)** in 8-day post-fertilization (dpf) medaka embryos, as shown as merged images in **(C,D)**. Scale bar is 10 μm.

IHC was applied with a medaka NHE2-specific antibody to localize NHE2 protein in ionocytes of SW acclimated embryos (Figure 5). NKA immunostaining was used as a marker of ionocytes. Confocal images showed that NHE2 signals (green; Figures 5A,C) were colocalized with NKA signals in the basolateral membrane of ionocytes (red; Figures 5B,C). The basolateral localization of NHE2 was also confirmed by the confocal z-scanning images (Figure 5D).

### Mortality Rate and Morphology of Embryos Injected With Traditional or Photo-Activated Morpholino Oligonucleotides

**FIGURE 6:**
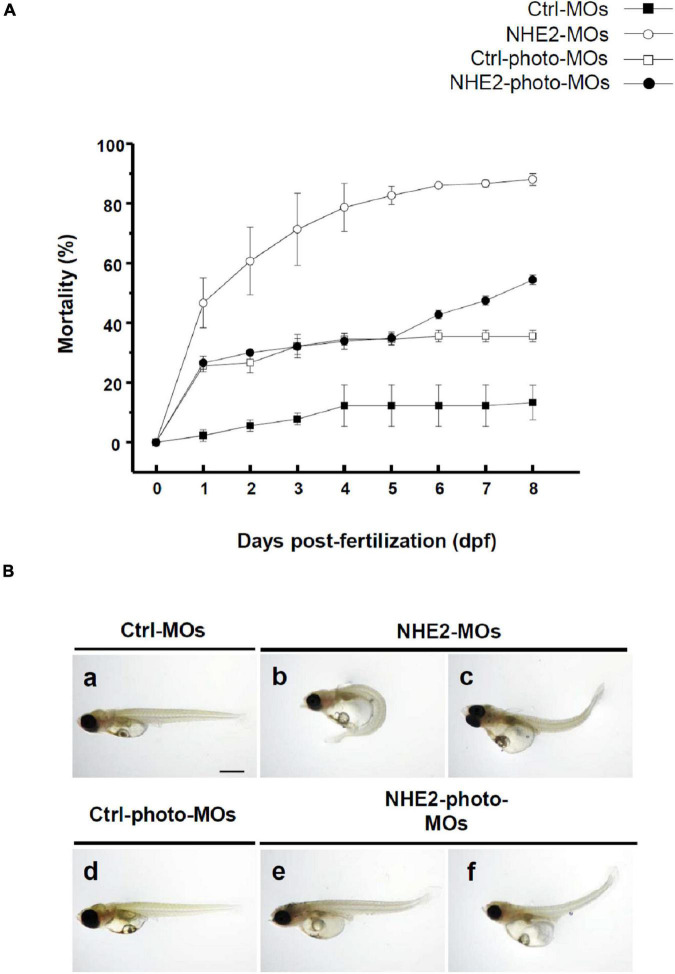
Effects of Na^+^/H^+^ exchanger 2 (NHE2)-knockdown on mortality and phenotype in seawater (SW)-acclimated embryos. Mortality of control morpholino oligonucleotides (Ctrl-MOs), NHE2-MOs, Ctrl-photoactivated (photo)-MOs, and NHE2-photo-MOs at 0-day post-fertilization (dpf) to 8-dpf SW-acclimated embryos **(A)**. Phenotypes of Ctrl-MOs, NHE2-MOs, Ctrl-photo-MOs, and NHE2-photo-MOs of 8-dpf SW-acclimated embryos **(B)**. Scale bar is 200 μm.

To knockdown NHE2 protein expression, traditional MOs were injected into embryos at the 2-cell stage. Injection with a low dose of NHE2-MOs (0.5 ng/embryo) dramatically increased the mortality rate to 83% at 5 dpf which reached 90% at 8 dpf (Figure 6A), while control-MOs showed only 12% mortality at 8 dpf. We also observed severe deformation in embryos, including edema and tail bending (Figure 6B), suggesting that NHE2 might be required for early embryo development. To reduce the impact on early development, a photo-MO technique was applied to knock-down NHE2 expression at a later stage (5 dpf). Results showed that the mortality of NHE2-photo-MOs was about 35% at 5 dpf and gradually increased to 55% at 8 dpf, while control-photo-MOs only caused 35% mortality at 8 dpf. Morphological defects were barely observed in NHE2-photo-MOs, with only slight edema or tail bending exhibited (Figure 6A).

**FIGURE 7:**
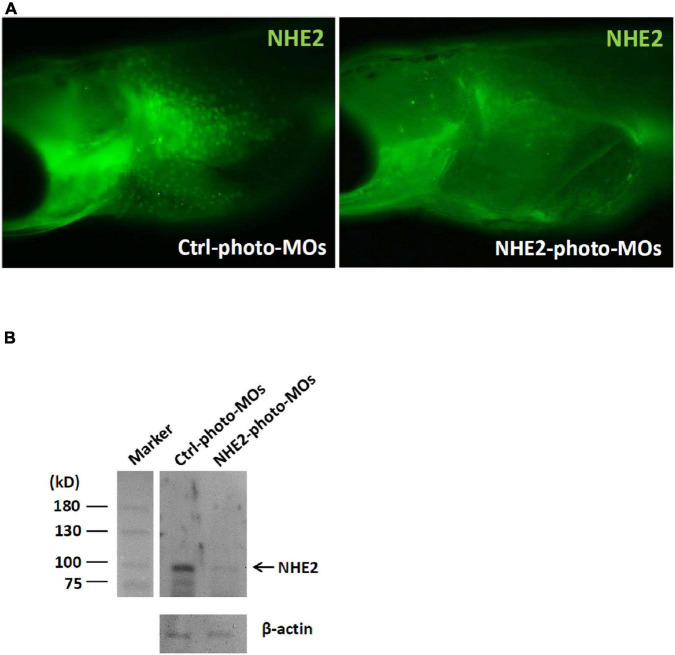
Na^+^/H^+^ exchanger 2 (NHE2) protein expression in seawater (SW)-acclimated medaka embryos. **(A)** Immunocytochemistry of NHE2 in 8-day post-fertilization (dpf) medaka embryos in the control (Ctrl) photoactivated morpholino oligonucleotide (photo-MO) and NHE2 photo-MO groups. **(B)** Western blot analysis of NHE2 in the Ctrl photo-MO and NHE2 photo-MO groups. β-Actin was used as an internal control.

An immunocytochemical analysis was used to compare NHE2 protein expression in 8-dpf embryos with or without NHE2-knockdown (KD). In the control-photo-MO group, green spots (NHE2 immunosignals) were observed on the yolk sac (Figure 7A), while those spots were not evident in the NHE2-photo-MO group. In addition, we also used a Western blot analysis to confirm NHE2-KD. Results showed a predicted band at 91 kDa in the control-photo-MO group, while the band was very faint in the NHE2-photo-MO group (Figure 7B).

### Effects of NHE2 Knockdown on the H^+^ Flux and Cl^–^ Flux of Ionocytes in Seawater-Acclimated Medaka Embryos

**FIGURE 8:**
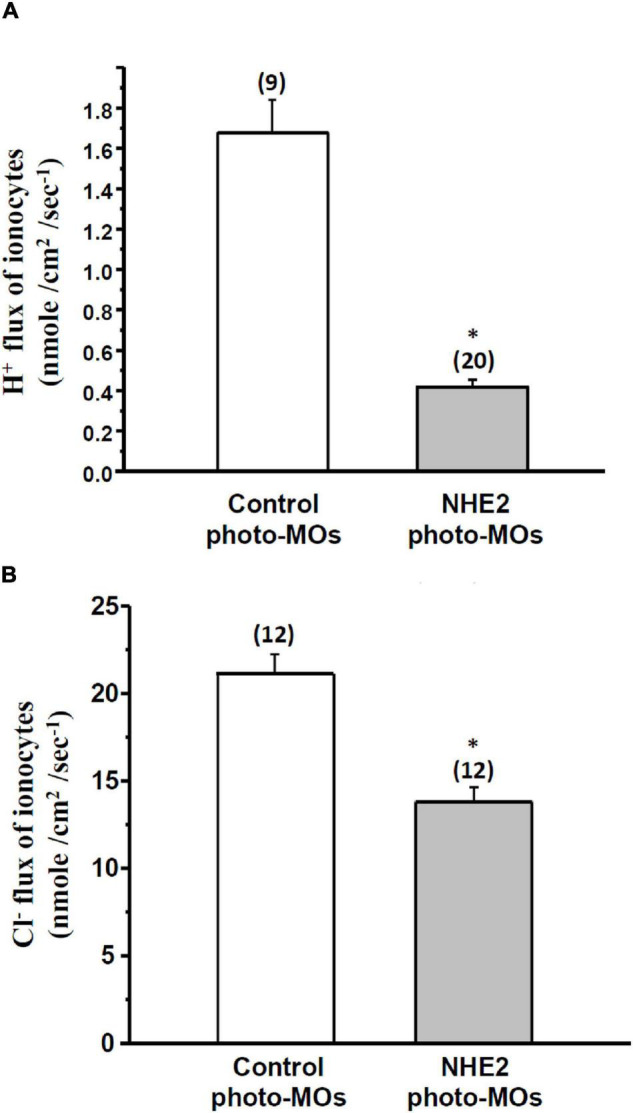
Effects of Na^+^/H^+^ exchanger 2 (NHE2)-knockdown on H^+^ flux and Cl^–^ flux of ionocytes in seawater (SW)-acclimated medaka embryos. Effects of control photoactivated morpholino oligonucleotides (Ctrl-photo-MOs) and NHE2 photo-MOs on H^+^ flux **(A)** and Cl^–^ flux **(B)** of ionocytes in medaka embryos acclimated to SW. Data are presented as the mean ± SE. The number of cells or larvae is shown in parentheses. * Indicates a significant difference (unpaired two-tailed Student’s *t-test*, *p* < 0.05).

To analyze the effects of NHE2 knockdown on H^+^ and Cl^–^ secretion by ionocytes, the SIET was used to measure H^+^ and Cl^–^ effluxes at individual ionocytes on the yolk sac skin of medaka. Results showed that the H^+^ efflux and Cl^–^ efflux significantly decreased by 60 and 34%, respectively, following NHE2 knockdown (Figures 8A,B).

## Discussion

According to the phylogenetic analysis (Figure 1), the fish NHE2 clade clustered with mammalian NHE2 and NHE4. This finding is consistent with previous findings that NHE2 of mummichog (*Fundulus heteroclitus*), dogfish (*Squalus acanthias*), and sculpin (*Myoxocephalus octodecemspinosus*) were similar to rat NHE2 and NHE4 (Claiborne et al., 2002). Furthermore, genomic loci of the physical distance (according to information collected from the Ensembl database) showed that *NHE2* and its adjacent genes, including *TMEM182* and *MFSD9*, were evolutionarily located on the same chromosome from fish to mammals. The *NHE4* gene appeared beside *NHE2* on the same chromosome of amphibians, reptiles, birds, and mammals but not in that of fishes, suggesting that *NHE4* might have been tandem-duplicated in amphibians from the proto *NHE2* gene (Figure 2).

Moreover, the full-length amino acid identities between the fish NHE2 clade and mammalian NHE2 clade were about 40.1∼44.7%, while those of the fish NHE2 clade and mammalian NHE4 clade were about 37.4∼41.9% (Supplementary Table 2), suggesting that the properties of fish NHE2 might be similar to those of both mammalian NHE2 and NHE4. In mammalian kidneys, NHE2 is mainly expressed in the apical membrane of the thick ascending limb (TAL), distal convoluted tubules (DCTs) and connecting tubules (CTs), while NHE4 is expressed in the basolateral membrane of the TAL and DCTs (Chambrey et al., 1998, 2001; Houillier and Bourgeois, 2012). The basolateral localization of medaka NHE2 is similar to that of mammalian NHE4. Chow and colleagues (Chow, 1999; Chow et al., 1999) identified an apical targeting signal in a 45-residue region of the cytosolic domain of mammalian NHE2. Deletion of the targeting region of NHE2 caused a miss-targeting of NHE2 to the basolateral membrane of renal cells. In the deduced protein sequences alignment of fishes to mammals, the apical targeting sequence of mammals was not found in fish NHE2 (Figure 3), suggesting that fish NHE2 is probably not an apical protein.

It is generally accepted that SW fishes excrete metabolic acid *via* NHEs, including NHE2 and NHE3, in the apical membrane of ionocytes (Evans et al., 2005; Hwang et al., 2011). However, previous studies did not provide convincing data to demonstrate the subcellular localization (apical or basolateral side) of NHE2 in SW fishes. Using heterologous antibodies (against mammalian NHE2), Edwards and colleagues localized NHE2 to ionocytes in gills of elasmobranches (Edwards et al., 2002). However, the subcellular localization of NHE2 was uncertain because granular signals were found in the cytoplasm of ionocytes (Edwards et al., 2002). In longhorn sculpin (*Myoxocephalus octodecemspinosus*), a homologous antibody was applied to localize NHE2 in ionocytes (Catches et al., 2006). They found only partial NHE2 signals in the apical membrane but most signals in the cytoplasm of ionocytes. Our previous study showed that the NHE2 transcript and NHE3 protein were expressed in the same ionocytes in SW-acclimated medaka (Liu et al., 2013). However, the subcellular localization of NHE2 in medaka ionocytes was unclear. In the present study, we generated an isoform-specific antibody to demonstrate the basolateral localization of NHE2 in medaka. To our knowledge, this is the first study to reveal the basolateral localization of NHE2 in fish ionocytes and the first study to examine its function with a gene-knockdown technique.

To investigate the function of NHE2 in medaka, a loss-of-function approach with MOs knockdown was conducted. With a traditional MOs injection, we found that the mortality rate was very high (∼90%), indicating that NHE2 might play a critical role in early embryonic development. To prevent the lethal effects in early development, a photo-MOs technique (Tallafuss et al., 2012) was used to knock down NHE2 expression at a later developmental stage. With the photo-MOs technique, we activated antisense MOs with UV light to knock down NHE2 expression at 5 dpf, and the mortality rate of embryos at 8 dpf was relatively lower (55%). In addition, the NHE2 protein abundance was successfully suppressed demonstrating the feasibility of the photo-MOs technique in medaka embryos. Using the SIET to analyze functional changes of ionocytes in NHE2 knockdown embryos, we found that both H^+^ and Cl^–^ excretion by ionocytes were suppressed (Figures 8A,B), suggesting that basolateral NHE2 plays a role in the H^+^ and Cl^–^ secretion.

Our previous study (Liu et al., 2013) compared the mRNA level of NHE3 and NHE2 in medaka acclimated to FW and SW. We found that NHE3 level was 4.5-fold higher in FW than in SW, suggesting that apical NHE3 is probably required for acid secretion in both conditions (the driving force for NHE is higher in SW). In contrast, NHE2 level was much lower in FW than in SW(19-fold lower), suggesting that basolateral NHE2 is specific for SW acclimation. The presence of NHE2 in SW ionocytes might be tightly associated with C1^–^ secretion which is specific for SW acclimation.

**FIGURE 9:**
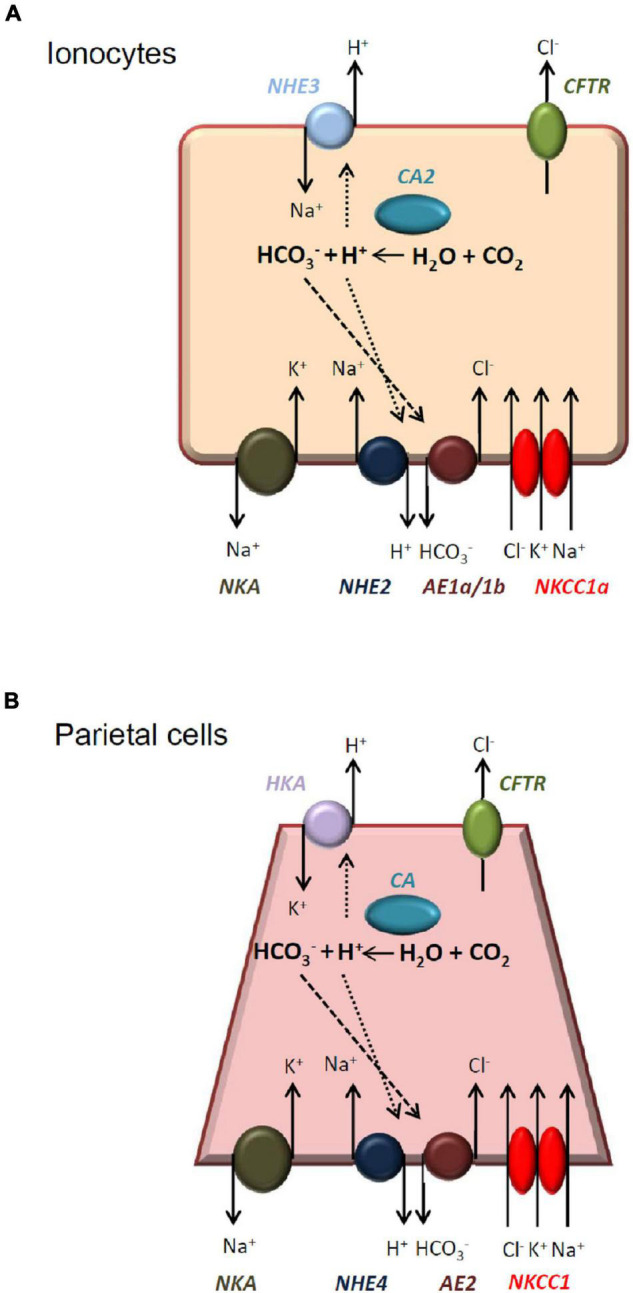
Illustrated models of the acid-secretion by medaka ionocytes and mammalian parietal cells. Medaka ionocytes **(A)** and mammalian parietal cells **(B)** are both acid-secreting cells and have similar transport mechanisms. See “Discussion” for a detailed comparison. NKA, Na^+^-K^+^-ATPase; NHE, Na^+^/H^+^ exchanger; AE, anion exchanger; NKCC, Na^+^-K^+^-Cl^–^ cotransporter; CA, carbonic anhydrase; HKA, H^+^/K^+^-ATPase; CFTR, cystic fibrosis transmembrane conductance regulator.

Our previous study demonstrated that ionocytes in the skin of SW-acclimated medaka are responsible for Cl^–^ and H^+^ secretion and proposed a mechanism for the linkage of Cl^–^ and H^+^ secretion (Liu et al., 2016). The basolateral localized NHE2 was probably involved in the mechanism and thus both Cl^–^ and H^+^ secretions were suppressed in the NHE2 knockdown embryos. Herein we proposed a modified model (Figure 9) for the possible role of NHE2 in ionocytes of medaka. In this model, anion exchanger 1(AE1), carbonic anhydrase 2 (CA2) and NHE2 might form a metabolon for HCO_3_^–^ reclaimation and Cl^–^ secretion by ionocytes. Cytosolic CA2 catalyzes CO_2_ hydration and produces H^+^ and HCO_3_^–^ intracellularly, and H^+^ and HCO_3_^–^ are, respectively, transported out of ionocytes *via* apical NHE3, basolateral NHE2 and basolateral AE1. In the basolateral membrane, an Na^+^ gradient provides the driving force for NHE2 to transport H^+^ out of the cell, while the H^+^ gradient facilitates Cl^–^/HCO_3_^–^ exchange by AE1. This proposed model is similar to the model (Figure 9) of gastric H^+^ secretion by mammalian parietal cells of gastric glands (Muallem and Sachs, 1985; Muallem et al., 1988; Gawenis et al., 2004). In parietal cells, basolateral NHE4 was suggested to be functionally associated with AE2 to promote Cl^–^ and HCO_3_^–^ exchange (Gawenis et al., 2005).

In conclusion, the present study demonstrated that NHE2 is expressed in the basolateral membrane of ionocytes in SW-acclimated medaka. Lost-of-function experiments with photo-activated morpholino oligonucleotides suggested that NHE2 is involved in H^+^ and Cl^–^ secretions by ionocytes. NHE2 and other basolateral transporters (such as AE1) might form a metabolon for HCO_3_^–^ reclaimation and Cl^–^ secretion by ionocytes of SW fishes. In the future, it is necessary to examine if the presented model (Figure 9) can be applied to other SW teleosts.

## Data Availability Statement

The raw data supporting the conclusions of this article will be made available by the authors, without undue reservation.

## Ethics Statement

The animal study was reviewed and approved by Animal Care and Utilization Committee of National Taiwan Normal University.

## Author Contributions

S-TL: conceptualization, methodology, writing—original draft preparation, investigation, and data curation. J-LH: conceptualization, methodology, formal analysis, and writing—reviewing and editing. L-YL: conceptualization, methodology, formal analysis, writing—reviewing and editing, and funding acquisition. All authors contributed to the article and approved the submitted version.

## Conflict of Interest

The authors declare that the research was conducted in the absence of any commercial or financial relationships that could be construed as a potential conflict of interest.

## Publisher’s Note

All claims expressed in this article are solely those of the authors and do not necessarily represent those of their affiliated organizations, or those of the publisher, the editors and the reviewers. Any product that may be evaluated in this article, or claim that may be made by its manufacturer, is not guaranteed or endorsed by the publisher.

## Footnotes

1
http://rsbweb.nih.gov/ij/download.html

2
http://www.cbs.dtu.dk/services/TMHMM/

## References

[B1] Catches J. S. Burns J. M. Edwards S. L. Claiborne J. B. (2006). Na^+^/H^+^ antiporter, V-H^+^-ATPase and Na^+^/K^+^-ATPase immunolocalization in a marine teleost (*Myoxocephalus octodecemspinosus*). *J. Exp. Biol.* 209 3440–3447. 10.1242/jeb.02384 16916979

[B2] Chambrey R. St John P. L. Eladari D. Quentin F. Warnock D. G. Abrahamson D. R. (2001). Localization and functional characterization of Na^+^/H^+^ exchanger isoform NHE4 in rat thick ascending limbs. *Am. J. Physiol. Renal Physiol.* 281 F707–F717. 10.1152/ajprenal.2001.281.4.F707 11553518

[B3] Chambrey R. Warnock D. G. Podevin R. A. Bruneval P. Mandet C. Bélair M. F. (1998). Immunolocalization of the Na^+^/H^+^ exchanger isoform NHE2 in rat kidney. *Am. J. Physiol. Physiol.* 275 F379–F386. 10.1152/ajprenal.1998.275.3.F379 9729510

[B4] Chow C. W. (1999). Regulation and intracellular localization of the epithelial isoforms of the Na^+^/H^+^ exchangers NHE2 and NHE3. *Clin. Invest. Med.* 22 195–206. 10.1016/S0016-5085(98)81606-5 10579058

[B5] Chow C. W. Woodside M. Demaurex N. Yu F. H. Plant P. Rotin D. (1999). Proline-rich motifs of the Na^+^/H^+^ exchanger 2 isoform. Binding of Src homology domain 3 and role in apical targeting in epithelia. *J. Biol. Chem.* 274 10481–10488. 10.1074/jbc.274.15.10481 10187839

[B6] Claiborne J. B. Blackston C. R. Choe K. P. Dawson D. C. Harris S. P. Mackenzie L. A. (1999). A mechanism for branchial acid excretion in marine fish: identification of multiple Na^+^/H^+^ antiporter (NHE) isoforms in gills of two seawater teleosts. *J. Exp. Biol.* 202 315–324. 10.1111/j.1095-8649.2009.02534.x 9882643

[B7] Claiborne J. B. Edwards S. L. Morrison-Shetlar A. I. (2002). Acid-base regulation in fishes: cellular and molecular mechanisms. *J. Exp. Zool.* 293 302–319. 10.1002/jez.10125 12115903

[B8] Cooper C. A. Wilson J. M. Wright P. A. (2013). Marine, freshwater and aerially acclimated mangrove rivulus (*Kryptolebias marmoratus*) use different strategies for cutaneous ammonia excretion. *Am. J Physiol. Regul. Integr. Comp. Physiol.* 304 R599–R612. 10.1152/ajpregu.00228.2012 23389109 PMC3627952

[B9] Edwards S. L. Donald J. A. Toop T. Donowitz M. Tse C. M. (2002). Immunolocalisation of sodium/proton exchanger-like proteins in the gills of elasmobranchs. *Comp. Biochem. Physiol. A Mol. Integr. Physiol.* 131 257–265. 10.1016/S1095-6433(01)00449-4 11818215

[B10] Edwards S. L. Wall B. P. Morrison-Shetlar A. Sligh S. Weakley J. C. Claiborne J. B. (2005). The effect of environmental hypercapnia and salinity on the expression of NHE-like isoforms in the gills of a euryhaline fish (*Fundulus heteroclitus*). *J. Exp. Zool. A Comp. Exp. Biol.* 303 464–475. 10.1002/jez.a.175 15880778

[B11] Evans D. H. Piermarini P. M. Choe K. P. (2005). The multifunctional fish gill: dominant site of gas exchange, osmoregulation, acid-base regulation, and excretion of nitrogenous waste. *Physiol. Rev.* 85 97–177. 10.1152/physrev.00050.2003 15618479

[B12] Gawenis L. R. Gawenis L. R. Greeb J. M. Prasad V. Grisham C. Sanford L. P. (2005). Impaired gastric acid secretion in mice with a targeted disruption of the NHE4 Na^+^/H^+^ exchanger. *J. Biol. Chem.* 280 12781–12789. 10.1074/jbc.M414118200 15684419

[B13] Gawenis L. R. Hut H. Bot A. G. Shull G. E. de Jonge H. R. Stien X. (2004). Electroneutral sodium absorption and electrogenic anion secretion across murine small intestine are regulated in parallel. *Am. J. Physiol. Gastrointest. Liver Physiol.* 287 G1140–G1149. 10.1152/ajpgi.00177.2004 15284023

[B14] Houillier P. Bourgeois S. (2012). More actors in ammonia absorption by the thick ascending limb. *Am. J. Physiol. Renal Physiol.* 302 F293–F297. 10.1152/ajprenal.00307.2011 22088435

[B15] Hu M. Y. Lee J. R. Lin L. Y. Shih T. H. Stumpp M. Lee M. F. (2013). Development in a naturally acidified environment: Na^+^/H^+^-exchanger 3-based proton secretion leads to CO_2_ tolerance in cephalopod embryos. *Front. Zool.* 10:51. 10.1186/1742-9994-10-51 23988184 PMC3844404

[B16] Hwang P. P. Chou M. Y. (2013). Zebrafish as an animal model to study ion homeostasis. *Pflügers Arch.* 465 1233–1247. 10.1007/s00424-013-1269-1 23568368 PMC3745619

[B17] Hwang P. P. Lee T. H. Lin L. Y. (2011). Ion regulation in fish gills: recent progress in the cellular and molecular mechanisms. *Am. J. Physiol. Regul. Integr. Comp. Physiol.* 301 R28–R47. 10.1152/ajpregu.00047.2011 21451143

[B18] Inokuchi M. Hiroi J. Watanabe S. Lee K. M. Kaneko T. (2008). Gene expression and morphological localization of NHE3, NCC and NKCC1a in branchial mitochondria-rich cells of *Mozambique tilapia* (*Oreochromis mossambicus*) acclimated to a wide range of salinities. *Comp. Biochem. Physiol. A Mol. Integr. Physiol.* 151 151–158. 10.1016/j.cbpa.2008.06.012 18619551

[B19] Inoue D. Wittbrodt J. (2011). One for all–a highly efficient and versatile method for fluorescent immunostaining in fish embryos. *PLoS One* 6:e19713. 10.1371/journal.pone.0019713 21603650 PMC3094454

[B20] Liu S. T. Horng J. L. Chen P. Y. Hwang P. P. Lin L. Y. (2016). Salt secretion is linked to acid-base regulation of ionocytes in seawater-acclimated medaka: new insights into the salt-secreting mechanism. *Sci. Rep.* 6:31433. 10.1038/srep31433 27511107 PMC4980601

[B21] Liu S. T. Tsung L. Horng J. L. Lin L. Y. (2013). Proton-facilitated ammonia excretion by ionocytes of medaka (*Oryzias latipes*) acclimated to seawater. *Am. J. Physiol. Regul. Integr. Comp. Physiol.* 305 R242–R251. 10.1152/ajpregu.00047.2013 23678031

[B22] Muallem S. Sachs G. (1985). Ca^2+^ metabolism during cholinergic stimulation of acid secretion. *Am. J. Physiol.* 248 G216–G228. 10.1152/ajpgi.1985.248.2.G216 3918459

[B23] Muallem S. Blissard D. Cragoe E. J. Jr. Sachs G. (1988). Activation of the Na^+^/H^+^ and Cl^–^/HCO_3_- exchange by stimulation of acid secretion in the parietal cell. *J. Biol. Chem.* 263 14703–14711. 3170561

[B24] Seo M. Y. Mekuchi M. Teranishi K. Kaneko T. (2013). Expression of ion transporters in gill mitochondrion-rich cells in Japanese eel acclimated to a wide range of environmental salinity. *Am. J Physiol. Regul. Integr. Comp. Physiol.* 166 323–332. 10.1016/j.cbpa.2013.07.004 23838143

[B25] Shen W. P. Horng J. L. Lin L. Y. (2011). Functional plasticity of mitochondrion-rich cells in the skin of euryhaline medaka larvae (*Oryzias latipes*) subjected to salinity changes. *Am. J Physiol. Regul. Integr. Comp. Physiol.* 300 R858–R868. 10.1152/ajpregu.00705.2010 21191003

[B26] Takeda H. Shimada A. (2010). The art of medaka genetics and genomics: what makes them so unique ? *Annu. Rev. Genet.* 44 217–241. 10.30047/JGMB.199103.0005 20731603

[B27] Tallafuss A. Gibson D. Morcos P. Li Y. Seredick S. Eisen J. (2012). Turning gene function ON and OFF using sense and antisense photo-morpholinos in zebrafish. *Development* 139 1691–1699. 10.1242/dev.072702 22492359 PMC3317972

[B28] Tresguerres M. Katoh F. Fenton H. Jasinska E. Goss G. G. (2005). Regulation of branchial V-H^+^-ATPase, Na^+^/K^+^-ATPase and NHE2 in response to acid and base infusions in the Pacific spiny dogfish (*Squalus acanthias*). *J. Exp. Biol.* 208 345–354. 10.1242/jeb.01382 15634853

[B29] Wittbrodt J. Shima A. Schartl M. (2002). Medaka–a model organism from the far East. *Nat. Rev. Genet.* 3 53–64. 10.1038/nrg704 11823791

[B30] Wu S. C. Horng J. L. Liu S. T. Hwang P. P. Wen Z. H. Lin C. S. (2010). Ammonium-dependent sodium uptake in mitochondrion-rich cells of medaka (*Oryzias latipes*) larvae. *Am. J. Physiol. Cell Physiol.* 298 C237–C250. 10.1152/ajpcell.00373.2009 19940071

[B31] Yan J. J. Chou M. Y. Kaneko T. Hwang P. P. (2007). Gene expression of Na^+^/H^+^ exchanger in zebrafish H^+^ -ATPase-rich cells during acclimation to low-Na^+^ and acidic environments. *Am. J. Physiol. Cell Physiol.* 293 C1814–C1823. 10.1152/ajpcell.00358.2007 17913841

[B32] Yan J. J. Hwang P. P. (2019). Novel discoveries in acid-base regulation and osmoregulation: a review of selected hormonal actions in zebrafish and medaka. *Gen. Comp. Endocrinol.* 277 20–29. 10.1016/j.ygcen.2019.03.007 30878350

