# Supplementary material for: Role of the Basolateral Na+/H+ Exchanger-2 (NHE2) in Ionocytes of Seawater- Acclimated Medaka (Oryzias latipes)
Source: Front Physiol. 2022 Mar 24;13:870967. doi: 10.3389/fphys.2022.870967 (PMC8987715; doi:10.3389/fphys.2022.870967)
Supplement: Supplementary file 1 [file Data_Sheet_1.doc]

**Supplemental Table 1.** **Summary of the known and predicted homologs of solute carrier 9A (SLC9A) protein family. Gene loci organization in the genomic region are collected from ENSEMBL Genome Browser.**

| **SLC9A1 orthologs** | | | | | |
| --- | --- | --- | --- | --- | --- |
| **Gene name** | **Protein** | **Species** | **Gene loci** | **Accession/Prediction Numbers** | |
| hsSLC9A1 | hsNHE1 | *Homo sapiens* | Ch.1: 27.4m | NP_003038.2 | |
| mmSLC9A1 | mmNHE1 | *Mus musculus* | Ch.4: 133.3m | NP_058677.1 | |
| rnSLC9A1 | rnNHE1 | *Rattus norveqicus* | Ch.5: 155.2m | ENSRNOP00000011049 | |
| ggSLC9A1 | ggNHE1 | *Gallus gallus* | Ch.23: 1.85m | NP_001038108.1 | |
| xlSLC9A1 | xtNHE1 | *Xenopus_laevis* | Scaffold GL172742.1: 2.12m | NP_001081553.1 | |
| drSLC9A1 | drNHE1 | *Danio rerio* | Ch.16: 55.1m | NP_001106952.1 | |
| **olSLC9A1a** | **olNHE1a** | ***Oryzias latipes*** | **Ch.16: 21.7m** | **XP_004078493.1** | |
| **olSLC9A1b** | **olNHE1b** | ***Oryzias latipes*** | **Ch.11: 16.7m** | **ENSORLP00000009662** | |
| trSLC9A1a | trNHE1a | *Takifugu rubripes* | Scaffold 35: 423 .8k | ENSTRUP00000018461 | |
| trSLC9A1b | trNHE1b | *Takifugu rubripes* | Scaffold 45: 147.9m | ENSTRUP00000015472 | |
| **SLC9A2 orthologs** | | | | | |
| **Gene name** | **Protein** | **Species** | **Gene loci** | | **Accession/Prediction Numbers** |
| hsSLC9A2 | hsNHE2 | *Homo sapiens* | Ch.2: 103.2m | | NP_003039.2 |
| mmSLC9A2 | mmNHE2 | *Mus musculus* | Ch.1: 40.6m | | NP_001028461.2 |
| rnSLC9A2 | rnNHE2 | *Rattus norveqicus* | Ch.9: 47.06m | | NP_001106806.1 |
| ggSLC9A2 | ggNHE2 | *Gallus gallus* | Ch.1: 133.3m | | NP_001272864.1 |

| **SLC9A3 orthologs** | | | | |
| --- | --- | --- | --- | --- |
| **Gene name** | **Protein** | **Species** | **Gene loci** | **Accession/Prediction Numbers** |
| hsSLC9A3 | hsNHE3 | *Homo sapiens* | Ch.5: 473.4k | NP_004165.2 |
| mmSLC9A3 | mmNHE3 | *Mus musculus* | Ch.13: 74.1m | NP_001074529.1 |
| rnSLC9A3 | rnNHE3 | *Rattus norveqicus* | Ch.1: 33.1m | NP_036786.1 |
| ggSLC9A3 | ggNHE3 | *Gallus gallus* | Ch.2: 55.8m | ENSGALP00000020419 |
| xtSLC9A3 | xtNHE3 | *Xenopus tropicalis* | Scaffold GL172641.1: 4.65m | ENSXETP00000050086 |
| drSLC9A3a | drNHE3a | *Danio rerio* | Ch.19: 11.3m | ABU68834.1 |

(Continued)

| **SLC9A3 orthologs** | | | | |
| --- | --- | --- | --- | --- |
| **Gene name** | **Protein** | **Species** | **Gene loci** | **Accession/Prediction Numbers** |
| drSLC9A3b | drNHE3b | *Danio rerio* | Ch.19: 11.3m | ABU68830.1 |
| **olSLC9A3** | **olNHE3** | ***Oryzias latipes*** | **Ch.11: 18.7m** | **ENSORLP00000011453** |

| **SLC9A4 orthologs** | | | | |
| --- | --- | --- | --- | --- |
| **Gene name** | **Protein** | **Species** | **Gene loci** | **Accession/Prediction Numbers** |
| hsSLC9A4 | hsNHE4 | *Homo sapiens* | Ch.2: 103.08m | NP_001011552.2 |
| mmSLC9A4 | mmNHE4 | *Mus musculus* | Ch.1: 40.5m | NP_796058.1 |
| rnSLC9A4 | rnNHE4 | *Rattus norveqicus* | Ch.9: 46.9m | NP_775121.1 |
| ggSLC9A4 | ggNHE4 | *Gallus gallus* | Ch.1: 133.3m | ENSGALP00000035784 |
| xtSLC9A4 | xtNHE4 | *Xenopus tropicalis* | Scaffold GL172737.1: 426.6k | ENSXETP00000058572 |
| drSLC9A4 | drNHE4 | *Danio rerio* | Ch.9: 7.004m | [ENSDARP00000076157](http://asia.ensembl.org/Danio_rerio/Transcript/ProteinSummary?db=core;g=ENSDARG00000058780;r=9:7004555-7033166;t=ENSDART00000081718) |
| gaSLC9A4 | gaNHE4 | *Gasterosteus aculeatus* | groupXVI: 7.28m | ENSGACP00000003875 |
| **olSLC9A4** | **olNHE4** | ***Oryzias latipes*** | **Ch.21: 11.72m** | **ENSORLP00000015518** |
| trSLC9A4 | trNHE4 | *Takifugu rubripes* | Scaffold 42: 904k | ENSTRUP00000035096 |
| tnSLC9A4 | tnNHE4 | *Tetraodon nigroviridis* | Ch.2: 11.2m | ENSTNIP00000016881 |

| **SLC9A5 orthologs** | | | | |
| --- | --- | --- | --- | --- |
| **Gene name** | **Protein** | **Species** | **Gene loci** | **Accession/Prediction Numbers** |
| hsSLC9A5 | hsNHE5 | *Homo sapiens* | Ch.16: 67.2m | NP_004585.1 |
| mmSLC9A5 | mmNHE5 | *Mus musculus* | Ch.8: 105.3m | NP_001074801.1 |
| rnSLC9A5 | rnNHE5 | *Rattus norveqicus* | Ch.19: 48.1m | NP_620213.1 |
| ggSLC9A5 | ggNHE5 | *Gallus gallus* | Ch.11: 1.32m | ENSGALP00000012759 |
| xtSLC9A5 | xtNHE5 | *Xenopus tropicalis* | Scaffold GL172641.1: 4.65m | ENSXETP00000017713 |
| drSLC9A5 | drNHE5 | *Danio rerio* | Ch.7: 36.07m | NP_001106943.1 |
| **olSLC9A5** | **olNHE5** | ***Oryzias latipes*** | **Ultracontig49: 423k** | **ENSORLP00000023205** |

(Continued)

| **SLC9A6 orthologs** | | | | |
| --- | --- | --- | --- | --- |
| **Gene name** | **Protein** | **Species** | **Gene loci** | **Accession/Prediction Numbers** |
| hsSLC9A6 | hsNHE6 | *Homo sapiens* | Ch.X: 135.06m | NP_001036002.1 |
| mmSLC9A6 | mmNHE6 | *Mus musculus* | Ch.X: 56.6m | NP_766368.2 |
| rnSLC9A6 | rnNHE6 | *Rattus norveqicus* | Ch.X: 153.6m | [ENSRNOP00000060014](http://asia.ensembl.org/Rattus_norvegicus/Transcript/Sequence_Protein?db=core;g=ENSRNOG00000000879;t=ENSRNOT00000066809) |
| ggSLC9A6 | ggNHE6 | *Gallus gallus* | Ch.4: 4.16m | ENSGALP00000009962 |
| xtSLC9A6 | xtNHE6 | *Xenopus tropicalis* | Scaffold GL172667.1: 1.4m | ENSXETP00000042778 |
| drSLC9A6a | drNHE6a | *Danio rerio* | Ch.14: 32.4m | ENSDARP00000014665 |
| drSLC9A6b | drNHE6b | *Danio rerio* | Ch.10: 27.2m | ABU68836.1 |
| **olSLC9A6a** | **olNHE6a** | ***Oryzias latipes*** | **Ch.10: 6.3m** | **ENSORLP00000001919** |
| **olSLC9A6b** | **olNHE6b** | ***Oryzias latipes*** | **Ch.14: 7.7m** | **ENSORLP00000002224** |

| **SLC9A7 orthologs** | | | | |
| --- | --- | --- | --- | --- |
| **Gene name** | **Protein** | **Species** | **Gene loci** | **Accession/Prediction Numbers** |
| hsSLC9A7 | hsNHE7 | *Homo sapiens* | Ch.X: 46.4m | NP_115980.1 |
| mmSLC9A7 | mmNHE7 | *Mus musculus* | Ch.X: 20.1m | NP_796327.1 |
| rnSLC9A7 | rnNHE7 | *Rattus norveqicus* | Ch.X: 3.22m | NP_001101712.1 |
| ggSLC9A7 | ggNHE7 | *Gallus gallus* | Ch.1: 129.6m | ENSGALP00000026953 |
| xtSLC9A7 | xtNHE7 | *Xenopus tropicalis* | Scaffold GL172889.1: 0.98m | NP_001120903.1 |
| drSLC9A7 | drNHE7 | *Danio rerio* | Ch.6: 37.4m | NP_001025248.2 |
| **olSLC9A7** | **olNHE7** | ***Oryzias latipes*** | **Ch.4: 30.3m** | **ENSORLP00000020073** |

| **SLC9A8 orthologs** | | | | |
| --- | --- | --- | --- | --- |
| **Gene name** | **Protein** | **Species** | **Gene loci** | **Accession/Prediction Numbers** |
| hsSLC9A8 | hsNHE8 | *Homo sapiens* | Ch.20: 48.4m | NP_056081.1 |
| mmSLC9A8 | mmNHE8 | *Mus musculus* | Ch.2: 167.4m | NP_848458.2 |
| rnSLC9A8 | rnNHE8 | *Rattus norveqicus* | Ch.3: 170.3m | NP_001020452.1 |
| ggSLC9A8 | ggNHE8 | *Gallus gallus* | Ch.20: 14.1m | NP_001034364.1 |

(Continued)

| **SLC9A8 orthologs** | | | | |
| --- | --- | --- | --- | --- |
| **Gene name** | **Protein** | **Species** | **Gene loci** | **Accession/Prediction Numbers** |
| xtSLC9A8 | xtNHE8 | *Xenopus tropicalis* | Scaffold GL172684.1: 1.36m | ENSXETP00000051782 |
| drSLC9A8 | drNHE8 | *Danio rerio* | Ch.23: 4.004m | ENSDARP00000027230 |
| **olSLC9A8** | **olNHE8** | ***Oryzias latipes*** | **Ch.7: 15.6m** | **ENSORLP00000011959** |

| **SLC9A9 orthologs** | | | | |
| --- | --- | --- | --- | --- |
| **Gene name** | **Protein** | **Species** | **Gene loci** | **Accession/Prediction Numbers** |
| hsSLC9A9 | hsNHE9 | *Homo sapiens* | Ch.3: 142.9m | NP_775924.1 |
| mmSLC9A9 | mmNHE9 | *Mus musculus* | Ch.9: 94.6m | NP_808577.3 |
| rnSLC9A9 | rnNHE9 | *Rattus norveqicus* | Ch.8: 101.7m | [ENSRNOP00000011358](http://asia.ensembl.org/Rattus_norvegicus/Transcript/Sequence_Protein?db=core;g=ENSRNOG00000008554;t=ENSRNOT00000011358) |
| ggSLC9A9 | ggNHE9 | *Gallus gallus* | Ch.9: 10.1m | ENSGALP00000039730 |
| xtSLC9A9 | xtNHE9 | *Xenopus tropicalis* | Scaffold GL173006.1: 0.69m | ENSXETP00000003421 |
| onSLC9A9 | onNHE9 | *Oreochromis niloticus* | Scaffold GL831138.1: 1.15m | ENSONIP00000007423 |

| **SLC9B1 orthologs** | | | | |
| --- | --- | --- | --- | --- |
| **Gene name** | **Protein** | **Species** | **Gene loci** | **Accession/Prediction Numbers** |
| hsSLC9B1 | hsNHA1 | *Homo sapiens* | Ch.4: 103.8m | ENSP00000296422 |
| mmSLC9B1 | mmNHA1 | *Mus musculus* | Ch.3: 135.3m | ENSMUSP00000077644 |
| rnSLC9B1 | rnNHA1 | *Rattus norveqicus* | Ch.2: 259.04m | ENSRNOP00000029131 |
| drSLC9B1a | drNHA1a | *Danio rerio* | Ch.1: 44.07m | ENSDARP00000069088 |
| drSLC9B1b | drNHA1b | *Danio rerio* | Ch.23: 44.1m | ENSDARP00000110731 |
| **olSLC9B1a** | **olNHA1a** | ***Oryzias latipes*** | **Ch.1:19.007m** | **ENSORLP 00000007692** |
| **olSLC9B1b** | **olNHA1b** | ***Oryzias latipes*** | **Ultracontig 203: 121.4k** | **ENSORLP00000023672** |

| **SLC9B2 orthologs** | | | | |
| --- | --- | --- | --- | --- |
| **Gene name** | **Protein** | **Species** | **Gene loci** | **Accession/Prediction Numbers** |
| hsSLC9B2 | hsNHA2 | *Homo sapiens* | Ch.4: 103.9m | ENSP00000378265 |

(Continued)

| **SLC9B2 orthologs** | | | | |
| --- | --- | --- | --- | --- |
| **Gene name** | **Protein** | **Species** | **Gene loci** | **Accession/Prediction Numbers** |
| mmSLC9B2 | mmNHA2 | *Mus musculus* | Ch.3: 135.3m | ENSMUSP00000060640 |
| rnSLC9B2 | rnNHA2 | *Rattus norveqicus* | Ch.2: 259.01m | ENSRNOP00000033064 |
| ggSLC9B2 | ggNHA2 | *Gallus gallus* | Ch.4: 60.5m | ENSGALP00000020101 |
| xtSLC9B2 | xtNHA2 | *Xenopus tropicalis* | Scaffold GL172747.1: 1.21m | ENSXETP00000046115 |
| drSLC9B2 | drNHA2 | *Danio rerio* | Ch.23: 44.2m | ENSDARP00000104800 |

| **SLC9C1 orthologs** | | | | |
| --- | --- | --- | --- | --- |
| **Gene name** | **Protein** | **Species** | **Gene loci** | **Accession/Prediction Numbers** |
| hsSLC9C1 | hsNHE10 | *Homo sapiens* | Ch.3: 111.8m | ENSP 00000306627 |
| mmSLC9C1 | mmNHE10 | *Mus musculus* | Ch.16: 45.5m | ENSMUSP 00000124969 |
| rnSLC9C1 | rnNHE10 | *Rattus norveqicus* | Ch.11: 64.2m | ENSRNOP 00000045235 |

| **SLC9C2 orthologs** | | | | |
| --- | --- | --- | --- | --- |
| **Gene name** | **Protein** | **Species** | **Gene loci** | **Accession/Prediction Numbers** |
| hsSLC9C2 | hsNHE11 | *Homo sapiens* | Ch.1: 173.4m | ENSP00000356687 |
| rnSLC9C2 | rnNHE11 | *Rattus norveqicus* | Ch.13: 83.8m | ENSRNOP00000030559 |

**Supplemental Table 2. Teleost NHE2 amino acid sequences among the identified mammalian NHE2 and NHE4 isoforms**


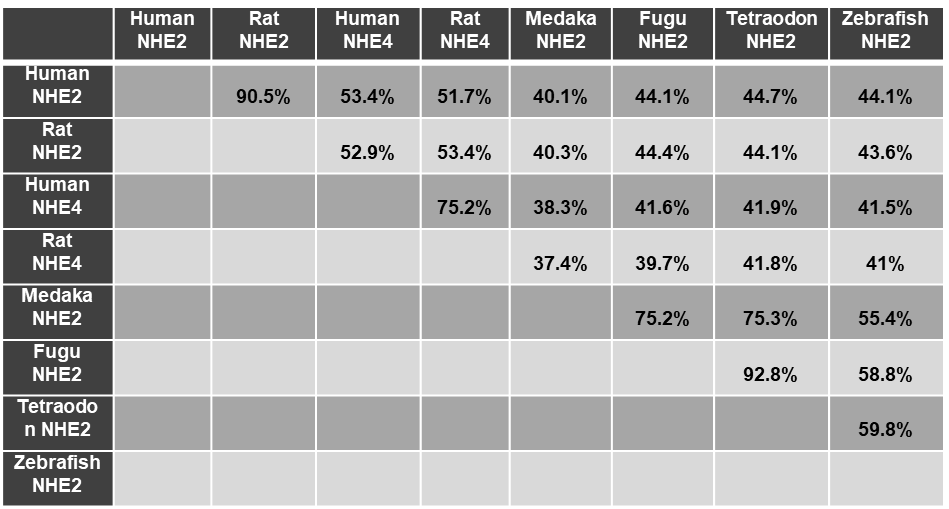


**Identity position (%)**
